# Advances in radiotherapy and comprehensive treatment of high-grade glioma: immunotherapy and tumor-treating fields

**DOI:** 10.7150/jca.51107

**Published:** 2021-01-01

**Authors:** Shiyu Liu, Qin Zhao, Weiyan Shi, Zhuangzhuang Zheng, Zijing Liu, Lingbin Meng, Lihua Dong, Xin Jiang

**Affiliations:** 1 Department of Radiation Oncology, The First Hospital of Jilin University, Changchun 130021, China; 2 Jilin Provincial Key Laboratory of Radiation Oncology & Therapy, The First Hospital of Jilin University, Changchun 130021, China; 3 NHC Key Laboratory of Radiobiology, School of Public Health, Jilin University, Changchun 130021, China; 4 Department of Hematology and Medical Oncology, Moffitt Cancer Center, Tampa, FL 33612, USA

**Keywords:** High-grade glioma, Immunotherapy, Molecular targeted drugs, Radiotherapy, Tumor-treating fields

## Abstract

High-grade gliomas (HGGs) are the most common primary malignant brain tumors. They have a high degree of malignancy and show invasive growth. The personal treatment plan for HGG is based on the patient's age, performance status, and degree of tumor invasion. The basic treatment plan for HGG involves tumor resection, radiotherapy (RT) with concomitant temozolomide (TMZ), and adjuvant TMZ chemotherapy. The basic radiation technology includes conventional RT, three-dimensional conformal RT, intensity-modulated RT, and stereotactic RT. As our understanding of tumor pathogenesis has deepened, so-called comprehensive treatment schemes have attracted attention. These combine RT with chemotherapy, molecular targeted therapy, immunotherapy, or tumor-treating fields. These emerging treatments are expected to improve the prospects of patients with HGG. In the present article, we review the recent advances in RT and comprehensive treatment for patients with newly diagnosed and recurrent HGG.

## Introduction

Glioma is the most common primary intracranial malignant tumor and accounts for 30% of all central nervous system tumors 1, 2. The World Health Organization (WHO) classification of central nervous system tumors places gliomas into grades I-IV, with grades I and II indicating low-grade gliomas and grades III and IV, high-grade gliomas (HGGs) 3. The incidence of HGGs is approximately 5 per 100,000 person-years. Researches have shown that gliomas are more common in adults and the elderly than in children; they also occur more often in men than in women 1, 2, 4, 5. HGGs are characterized by the "three high and one low" principle: high incidence rate, high recurrence rate, high mortality, and low cure rate.

HGG accounts for 2.4 percent of all cancer deaths. Although clinicians and researchers have endeavored to improve treatment, prognosis remains poor 6. After adjuvant chemotherapy, the 1-year survival rate of HGG patients is only 35% 7. Researchers have carried out several studies using more precise radiotherapy (RT), but the effect is still unsatisfactory 8. Therefore, new studies must be carried out to solve the difficulties associated with HGG treatment.

To treat newly diagnosed and recurrent HGG, RT generally involves conventional RT, three-dimensional conformal RT (3D-CRT), intensity-modulated RT (IMRT), and stereotactic RT (SRT). With a deeper understanding of the pathogenesis of HGG, a variety of comprehensive treatment modalities have been developed: RT has been combined with chemotherapy, molecular targeted therapy, and immunotherapy, while the tumor-treating fields (TTFields) approach has been combined with chemotherapy. These emerging treatment schemes can show better activity against tumors and improve survival rate. In this review, we focus on the progress of RT in the treatment of HGG.

## Radiotherapy

### Conventional radiotherapy

RT has a history of more than 100 years since its discovery in 1895, and it is now one of the three major treatment modalities for malignant tumors 9, some of which have such high sensitivity to radiation that use of RT alone is sufficient for cure. Examples of such tumors are nasopharyngeal carcinoma, early vocal fold cancer, and skin cancer. Conventional RT to treat HGG refers to two-dimensional conventional RT, which is applied to whole or part of the brain at an intensity of 45-60 Gy/25-30f 2, 10. Shibamoto et al.^11^ treated patients with HGG using conventional fractionation RT (64.8 Gy/36f) and demonstrated a median survival time of 14.5 months. Tanaka et al.^10^ treated 60 patients using conventional RT (60 Gy/30f) and observed an overall survival (OS) of 12.4 months. They noted that radiation-induced white-matter abnormality was more frequent with high-dose RT than with conventional RT. In a clinical trial by Bleehen et al.^12^, patients were divided into 45 Gy/20f and 60 Gy/20f groups, with 1-year survival rates of 29% and 39%, respectively. All these studies found that conventional RT showed good therapeutic effect in HGG, but the survival rate of patients following whole-brain irradiation was the same as that following tumor bed irradiation alone. So, irradiating whole brain is not necessary 13. In summary, although conventional segmentation can achieve a treatment effect, it also leads to many side effects; thus, it is necessary to improve the RT technology to achieve a better treatment effect.

### Three-dimensional conformal radiotherapy

3D-CRT applies a CT image to reconstruct the 3D structure of the tumor; the high-dose area is made coterminous with the lesion area to improve the radiation gain ratio and maximize the radiation dose within the lesion. Using 3D-CRT technology, tumor cells can be more effectively killed than when using conventional RT; furthermore, unnecessary irradiation of the surrounding normal tissues and organs can be reduced. Therefore, it is safe to increase the dose administered to the target tumor area, improve the local control rate of the tumor, improve survival, and reduce RT side effects. In the 1960s, Takahashi first proposed and clarified the basic concept and implementation method of conventional RT 14. Proimos et al. 15 improved this method and applied it to clinical practice. In the 1990s, 3D-CRT technology became the leading technological modality for tumor treatment. Lee et al.^16^ treated patients with HGG using 3D-CRT technology with a radiation dosage of 90 Gy. However, 3D-CRT alone is not sufficient, even at very high doses, and its local control is still poor; thus, for treatment HGG, 3D-CRT is usually combined with TMZ. This approach shows improved efficacy and safety after surgery. Thibouw et al.^17^ investigated patients with HGG who underwent 3D-CRT at a dosage of 40 Gy. They reported that the median survival was 13.4 months (range: 11.7-15.7 month). In conclusion, 3D-CRT is better than conventional RT, and 3D-CRT combined with TMZ confers more satisfactory results in HGG treatment.

### Intensity-modulated radiotherapy (IMRT)

IMRT is a new type of external RT technology that has been widely applied in clinical treatment. Both IMRT and 3D-CRT are first-choice RT techniques to treat HGG; using these techniques, clinicians can control the shape of the radiation field and the intensity of the RT machine to ensure that the internal region of the target area corresponds with its surface. Both 3D-CRT and IMRT can reduce the radiation dose to surrounding normal tissues and organs as well as increase the radiation dose in the tumor area. These RT techniques aim to protect normal tissues and kill the tumor 18.

IMRT shows better target coverage than 3D-CRT, and the dose required is lower when the tumor has a complex or irregular shape. In addition, it can reduce neurological toxicity as well as the dose applied to the optic nerve and other normal tissues 17, 19. Hermanto et al. 20 compared patients treated using 3D-CRT and IMRT. They found that the total integral dose on the normal tissues was reduced by 7%-10% using IMRT, that IMRT completed DNA breakage in tumor cells to promote cell death, and that it did not increase the dose or volume of normal tissue exposed to low doses of radiation. Cho et al. 21 treated HGG using simultaneous, integrated-boost IMRT (SIB-IMRT), with the following radiation dose: gross target volume, 60 Gy; clinical target volume, 50 Gy. The results showed that the median survival time was 14.8 months and the progression-free survival (PFS) time was 11 months. Thilmann et al. 22 treated HGG using IMRT and found that the treatment delivered a high dose to the enhancing lesion, and that the overall does was identical to that of an equivalent CTV dose delivered at the same time. Many clinical studies have shown that OS and PFS are higher in the IMRT group than in the conventional RT group, that treatment of HGG using low-segmentation and simultaneous IMRT shows satisfactory therapeutic effects, and that patients treated using this approach display good tolerance of adverse reactions. Importantly, IMRT has developed into a widely used RT technology; in fact, it is usually the first-choice RT in the treatment of HGG.

### Hyper-fractionated radiotherapy

Hyper-fractionated radiotherapy (HFRT) is a radiation technique that refers to reducing the dose of each irradiation twice a day or more, using a large number of smaller doses to make the total dose higher than the conventional dose. HFRT can improve the sublethal damage repair, has less dependence on oxygen, and increases the radiation opportunity of tumor cells in the more sensitive stage of the cell cycle 23. Prados et al. 24 treated HGG patients with 1.6 Gy each time, twice a day, and the total dose was 70.4 Gy. The results revealed that median survival period was 40 weeks and PFS period was 19 weeks. Nelson et al.^23^ treated HGG patients with 64.8, 72, 76.8, and 81.6 Gy, respectively, and observed that a total dose of 72 Gy/60f showed the best effect. Hasegawa et al. 25 transplanted xenografts of a human malignant glioma into nude mice and irradiated then with hyper-fractionated 24 Gy/20f (two fractions per day), which had a good effect on eliminating the tumor. Ali et al. 26 compared the patients who received conventional RT and HFRT. The results showed that the median survival time was 11.3 and 13.1 months, respectively. Xin et al. 27 conducted a comparative experiment and found that there was no clear difference in OS and PFS between the participants receiving HFRT and those receiving conventional RT. Although the dose administered in HFRT is higher than that given in conventional RT, there was no significant difference between the side effects and overall cognitive ability 28. Therefore, HFRT is not a necessary treatment technique for HGG.

### Stereotactic radiotherapy (SRT)

SRT uses stereotactic and multi-center, rotating irradiation technology to focus high-energy radiation in 3D space onto the limited target area of the lesion. It is characterized by high dose to the target area, large dose gradients with the surrounding normal tissues, and rapid tapering of radiation dose. However, SRT cannot cover the same area as conventional RT, so it cannot clear cells at the edge of the tumor and is therefore unsuitable as an initial treatment. Nonetheless, it is associated with a reduced reaction of the normal brain tissue to radiation, as well as with a decrease in complications, although it can cause vascular embolism, the death of proliferating cells, and marked necrosis within the treatment area. SRT has certain survival benefits for HGG patients with minimal disease burden 29. Shrieve et al.^30^ treated patients with HGG using SRT. They reported that the 12- and 24 month-OS rates were 88.5% and 35.9%, respectively, demonstrating that SRT has a survival advantage over other kinds of RT. Reynaud et al.^31^ investigated survival outcomes and safety in patients with recurrent HGG using HFSRT. They reported that HFSRT is feasible, with minimal adverse effects and a median OS of 15.6 months. In recent years, gamma knife stereotactic surgery has been applied in the rescue treatment of patients with recurrent HGG. This approach can improve the survival rate and reduce the burden 32. Overall, SRT should be considered for the treatment of recurrent HGG as it can reduce the treatment volume and decrease treatment-related toxicity, thus improving the safety of reirradiation 33.

## Combination therapy

### RT combined with chemotherapy

Chemotherapy can reduce the volume of lesions, improve local blood circulation, and increase radiosensitivity. Chemotherapy drugs have cell cycle-specific cytotoxic effects on S-phase cells, while concurrent RT and chemotherapy have complementary effects on cell killing. The most frequently used chemotherapy drug in HGG treatment is oral TMZ, which is widely distributed throughout the body without passing through the liver 6. It localizes easily to brain tumor cells after passing through the blood-brain barrier, and it acts on all cells in all stages of the cell cycle. Also, it shows rapid absorption and has a low incidence of side effects when administered orally 34.

The DNA repair enzyme O^6^-methylguanine-DNA methyltransferase (MGMT) inhibits the killing of tumor cells by TMZ. Methylation of the MGMT promoter silences this gene in cancer so that the cells no longer produce MGMT. This phenomenon is associated with tumor regression as well as with prolonged OS and disease-free survival 35, 36. The basic treatment regimen is to administer TMZ concurrently with radiation for 6 weeks, followed by 6 months of adjuvant TMZ therapy. However, because TMZ shows fair tolerability and because no effective second-line therapies are available, up to 12 cycles of treatment are now allowed. Roldan et al. 37 compared a treatment regimen of six cycles of monthly adjuvant TMZ with regimens involving more than six cycles. They found that the latter conferred significant benefits in terms of both PFS and OS. Hart et al. 38 conducted a randomized controlled trial and showed that TMZ increased both OS and PFS, but it was found to be associated with a significant impact on quality of life and increased the risk of hematological complications, fatigue, and infection. Stupp et al. 6 observed that patients with HGG treated using RT plus TMZ showed a 37% reduction in the risk of death compared with those who received RT alone. Jaymin et al. 39 treated one group of patients with HGG using chemotherapy alone and another group using chemotherapy plus conventional RT. They demonstrated no OS difference between the chemotherapy-alone and conventional RT groups. In elderly patients (≥65 years old) with newly diagnosed HGG, the addition of TMZ to short-course RT resulted in longer survival than short-course RT alone 40. Similarly, another chemotherapy treatment regimen is also available: procarbazine, lomustine, and vincristine (PCV). In several studies, PFS, but not OS, was better in patients with HGG treated using RT plus PCV than in those treated using RT alone, suggesting that chemotherapy has a delayed benefit 41, 42. One randomized trial revealed that primary chemotherapy was not superior to primary RT among patients with different kinds of glioma 43. Jakacki et al. 44 compared the survival rate between patients with glioma treated using lomustine plus TMZ and those treated using TMZ alone after conventional RT. They concluded that the addition of lomustine to TMZ as adjuvant therapy was associated with significantly better outcome. In our previous research, we concluded that RT plus chemotherapy is associated with considerable toxicity 45. In addition, RT can lead to secondary gliomas, and so other researchers have also considered combination therapy a potential avenue of treatment for radiation-induced gliomas 46. Overall, the combination of RT and chemotherapy is now the first choice of treatment for HGG.

### Molecular targeted drugs combined with RT

Currently, because glioma is resistant to RT and chemotherapy, it has a high recurrence rate and the treatment effect is not satisfying. To overcome these limitations of treatment, molecular targeted drugs combined with RT and chemotherapy have been researched. Molecular targeted drugs initiate specific molecular signal transduction pathways to kill the tumor effectively.

Molecular targeted therapies include vascular endothelial growth factor (VEGF) inhibitors and epidermal growth factor receptor (EGFR) inhibitors combined with RT 37, 47, 48. The signal transduction pathways involved may activate apoptosis, inhibit the cell cycle, or block vascular growth 49. Bevacizumab is a common molecular targeted drug; it is a humanized monoclonal antibody against VEGF 50 that can activate the VEGF downstream pathway (RAS-RAF-MAPK, etc.) through protein phosphorylation. In this manner, VEGF plays an important role in the growth and development of tumor cells 51. Bevacizumab is currently approved to treat patients with recurrent HGG, but not as part of the upfront regimen for newly diagnosed HGG 52. Friedman et al. 53 treated patients with HGG using 10 mg/kg bevacizumab, either alone or in combination with irinotecan. Both of these regimens produced satisfactory results in recurrent glioma. Hasselbalch et al. 54 observed that combining cetuximab with both bevacizumab and irinotecan was safe and effective in patients with recurrent HGG, that the median PFS was 16 weeks, and that the median OS was 30 weeks, which was an encouraging response rate. To treat patients with recurrent HGG, the combination of the anti-programmed cell death 1 (PD-1) monoclonal antibody nivolumab and bevacizumab showed no better efficacy than bevacizumab alone 55, 56. Sathornsumetee et al.^57^ demonstrated that bevacizumab combined with the EGFR tyrosine kinase inhibitor erlotinib was adequately tolerated in patients with recurrent HGG. Bovi et al. 58 compared PFS between two treatment strategies treating recurrent HGG patients and found that bevacizumab combined with RT, PFS was 12 months compared to bevacizumab alone where PFS was 4 months. Westphal et al. 37 conducted a clinical trial that failed to show any significant advantage of nimotuzumab in terms of prolonging PFS and OS when combined with bevacizumab to treat patients with HGG. Hofmann et al. 59 also utilized bevacizumab, citing improved survival in patients with recurrent HGG compared to standard therapy without bevacizumab (median OS, 10.3 vs. 4.2 months, p = 0.023). In a word, bevacizumab could improve clinical outcomes and the patient's quality of life in HGG treatment and show improvement in newly diagnosed and recurrent HGG patients.

Gefitinib is a potent small molecule inhibitor of EGFR tyrosine kinase. Schwer et al. 60 treated recurrent HGG using SRT combined with gefitinib and reported that a dose of 36 Gy in three fractions was well tolerated, with gefitinib at a daily dose of 250 mg. Aflibercept is a recombinant fusion protein of the VEGF extracellular domains. It binds with high affinity and can also scavenge VEGF 61. Groot et al. 62 evaluated its treatment efficacy in patients with recurrent HGG. They found that aflibercept monotherapy was not as effective as bevacizumab and that it had moderate toxicity. Bastiien et al.^63^ summarized some possible reasons for the failure of this molecular targeted therapy, which are as follows: (1) The targeted molecular pathways may have built-in redundancies; (2) It may be difficult to penetrate the blood-brain barrier to the central nervous system while avoiding neurotoxicity. Overall, RT combined with chemotherapy and molecular targeted drugs can lead to better treatment outcomes than the use of RT alone. This approach has become increasingly popular to treat recurrent HGG.

### Immunotherapy

**Figure 1:**
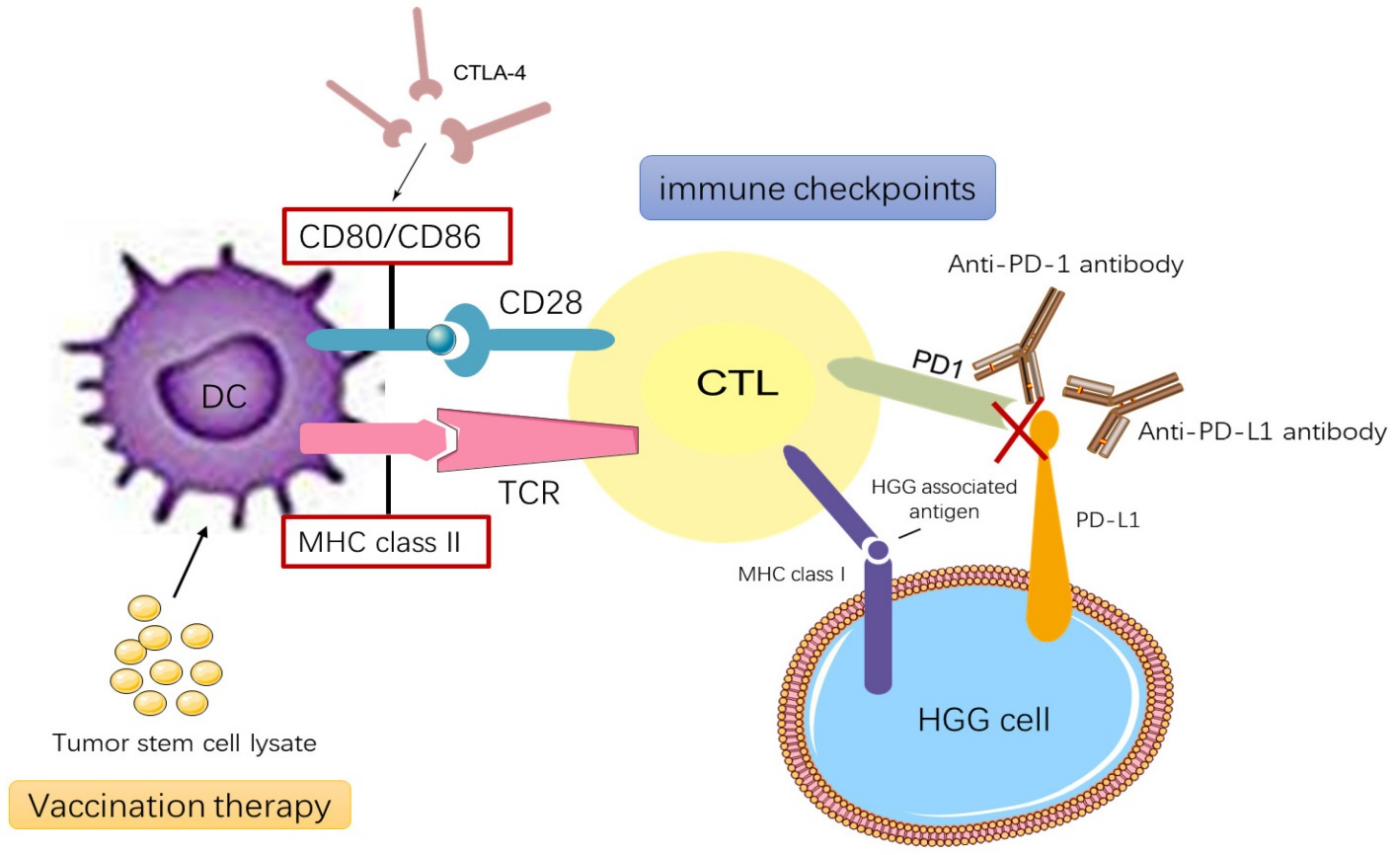
Current immunotherapy modalities for HGG treatments. HGG vaccination therapy relies on DC stimulated by tumor stem cell lysate or the interaction between MHC class II-TCR and CD80 and/or CD86-CD28. CTLs activate and destroy cells containing glioblastoma- associated antigens presented on MHC class I molecules. HGG cells upregulate the PD- L1 that combines with complementary receptors on the CTLs to cause inhibition of lymphocyte activation. CTLA-4 suppresses immune- checkpoint molecule that binds CD80 and CD86 and prevents their interaction with CD28.

Nowadays, immunotherapy is being applied to a broader range of diseases, and research is deepening our understanding of how immunotherapy could be used. The immune system can protect the host and inhibit the tumor microenvironment 64. Immunotherapy induces antitumor responses via the host immune system to clear tumors 65. RT releases tumor antigens and modulates immunological pathways, leading to increased tumor antigen concentration and major histocompatibility complex (MHC) on the tumor cell surface and priming of tumor-specific cytotoxic T cells, ultimately resulting in the immunogenic death of tumor cells 55, 66, 67. The primary target for the T-cell receptor is MHC loaded with tumor antigen 67. MHC I participates in antigen recognition by CD8^+^ cytotoxic T lymphocytes (CTLs), while MHC II participates in antigen recognition by CD4^+^ Th cell. The mechanisms of immunotherapy are depicted in Figure 1 68.

In recent years, antigen-specific cancer vaccines and immune checkpoint blockers has provided promising immunotherapeutic approaches to treat HGG 69. Two immune checkpoints being studied are cytotoxic T-lymphocyte-associated protein 4 (CTLA-4) and PD-1 70, 71, which along with programmed death-ligand 1 (PD-L1) is widely expressed in patients with either primary or recurrent HGG. The binding of PD-1 to PD-L1 induces apoptosis or leads to exhaustion of activated immune cells; therefore, blocking this interaction enhances antitumor activity, so patients can be treated using PD-1 and PD-L1 checkpoint inhibitors 72, 73. Through interactions with co-stimulatory molecules on other cells, CTLA-4 acts to decrease T-cell responsiveness 74. Tumor-associated macrophages (TAMs) contribute to tumor growth, metastasis, and neovascularization. In tumors trending towards malignancy, TAMs stimulate blood vessels and suppress antitumor immunity. HGG is characterized by extensive neo-angiogenesis, so TAMs are closely related to the pathogenesis of HGG 75, 76. Chimeric antigen receptor-T cell therapy integrates the target antigen receptor into the normal T cell gene through the carrier, while the target effector T cell is applied to the tumor site as an anti-tumor treatment.^77^ Scheetz et al.^69^ have developed a personalized nano-disc vaccine loaded with CpG—a toll-like receptor 9 agonist—as well as tumor-specific neoantigens that can be used in combination immunotherapy to treat recurrent gliomas. Zeng et al. 78 studied the efficacy of combination treatment using the anti-PD-1 antibody and RT in HGG mouse models. They demonstrated that this combination therapy resulted in long-term survival. Preclinical evidence suggests that hyper-fractionation RT can stimulate the immune system and make immunotherapy more effective 71. One mechanism accounting for the enhanced effectiveness of combinatorial treatment radiation-induced inflammation results in PD-L1 upregulation in cancer cells, macrophages, and dendritic cells 79. Fractionated RT leads to upregulation of PD-L1 expression in tumor cells, which generates therapeutic immune responses that can reduce tumor burden and improve survival 80. In conclusion, many studies have shown that immunotherapy can confer satisfactory therapeutic effects, especially when combined with molecular targeted therapy (Table 2).

### Tumor-treating fields (TTFields)

TTFields are an antimitotic treatment modality delivered via low-intensity, intermediate-frequency (200 kHz) alternating electric fields (≥ 18 hours/day) using four insulated transducer arrays placed directly on the skin in the region surrounding the tumor 81-84. Through dipole redistribution, this treatment acts on cells in the middle and late stages of mitosis (G_1_/S or G_2_/M), hinders the orderly arrangement and location of tubulin, disrupts the normal assembly of spindle microtubules, and leads to breakage of the centromere and damages the organelle structure^84^. The result is an overall decline in cell proliferation followed by apoptosis, which is strongly affected by mutations in p53. TTFields act on several molecular targets/pathways to influence Ca^2+^ and electrical signals. One recent study demonstrated that voltage-gated Ca^2+^ channel activity contributes to the cellular stress response to TTFields and that voltage-gated Ca^2+^ channel inhibition may augment the effects of TTFields 85. It thus follows that TTFields can reduce cell proliferation by specifically interfering with key proteins involved in cell division, leading to mitotic mutations and subsequent cell death 86.

**Figure 2:**
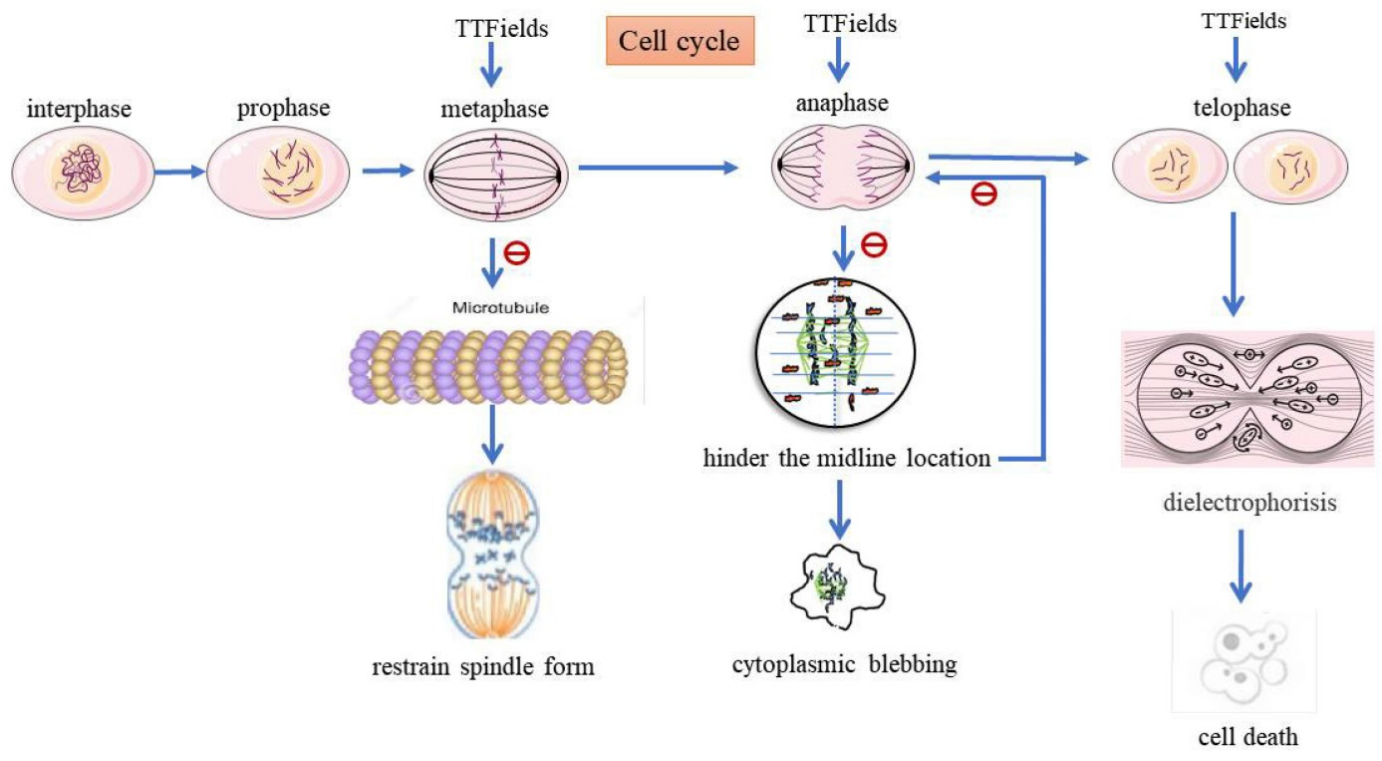
The mechanisms of TTFields treating HGG. TTFields will interfere with appropriate microtubules (MT) to prevent mitosis and spindle formation. TTFields exert directional force on MT, resulting in abnormal spindle formation, followed by stagnation or delay of mitosis. It may be caused by improper connection between chromosomes and spindle. TTFields suppress regulated BRCA / FANC gene, which make DNA replication fork pause and break down, and it can increase DNA damage and destroy DNA replication and repair mechanism of HGG cells.

The mechanisms of TTFields are depicted in Figure 2. TTFields can delay the repair of DNA damage caused in tumor cells by RT and chemotherapy, thus playing a role in killing tumor cells when used in coordination with RT and chemotherapy. This technique downregulates the *BRCA/FANC* genes and increases DNA single-/double-strand damage, thus destroying the DNA replication and repair mechanism in tumor cells. Because they act on hypoxic cells, TTFields inhibit mitochondrial autophagy in tumor cells, increase the production of reactive oxygen species, and improve the oxygen sensitivity.

When using TTFields, clinicians should adjust the transducer array layout according to the specific tumor location to improve the field strength and achieve the best effect of tumor cutting 87. And different TTFields intensities receive different results in PFS and OS for HGG patients 88. Many clinical trials have been carried out comparing TTFields plus TMZ with TMZ alone, and some trial has added lomustine to the treatment regimen, as shown in Table 3. Stupp et al. 82 treated patients with HGG using either TTFields plus TMZ or TMZ alone. They found that the median OS was 20.9 months and 16.0 months, respectively. The addition of TTFields to standard TMZ treatment in patients with HGG resulted in improved survival, with no negative impact on health-related quality of life other than itchy skin 89, 90. Furthermore, Lazaridis et al. [91]suggested that TTFields combined with lomustine and temozolomide was superior to TTFields combined with temozolomide monotherapy in patients with HGG. And this analysis provided first indication that the combination of TTFields/lomustine/temozolomide was safe and feasible. Lu et al. [92]compared PFS and OS between the two treatment strategies and found that the combination of TTFields, TMZ, bevacizumab, and irinotecan might play a more important role in the improvement of PFS and OS among HGG patients than the combination of TTFields and bevacizumab. Overall, TTFields combined with chemotherapy can prolong PFS and OS and is therefore the new pattern of treatment for patients with newly diagnosed or recurrent HGG 85, 93, 94.

## Conclusion

Considerable progress has been made in the field of RT in recent years, and it is important that clinicians and researchers maximize the survival benefits afforded by this treatment modality. At present, HGG is mainly treated using surgical resection combined with chemotherapy, RT, immunotherapy, and other comprehensive treatments. Postoperative external beam RT with concomitant TMZ and adjuvant TMZ chemotherapy has been recommended as a standard treatment for newly diagnosed HGG in adults. The typical RT dose is 60 Gy divided into 30 fractions. RT should be started as early as possible (2-6 weeks after surgery), and it can effectively prolong the survival period. The therapeutic effect of RT is cumulative, so it would not be effective if delivered all at one time. Instead, it takes effect if the patient accumulates a certain dose of radiation. Therefore, the exact irradiation time is not very strict—RT can be started as soon as possible provided a patient is prepared and a treatment plan has been established. In addition, age, pathological grade, and chemotherapy are related to the prognosis of HGG. Higher age is associated with a higher risk of death. There is an improved tend of prognosis with younger HGG patients. In addition, patients with low-grade gliomas have a better prognosis than those with HGG, and glioblastomas have the worst prognosis. Furthermore, postoperative concurrent chemoradiotherapy will further eliminate the tumor or inhibit the growth of the tumor, which will also improve the survival rate of HGG patients. Although these therapies represent a significant accomplishment, the treatment course is still riddled with numerous obstacles and challenges. RT may increase the risk of neurocognitive side effects in the long term, and the prognosis of HGG is still poor, so researchers face many challenges. As technology progresses, more exploration and research will be conducted, and more advanced technologies and approaches will be applied to the treatment of HGG in future. Furthermore, the diagnosis of HGG will become more accurate, and the therapeutic effect will be more refined.

## Figures and Tables

**Table 1:** Clinical trials of IMRT in HGG

Author	Total Dose (Gy)	Plan	Number of Patients	Result	Reference
Sutera et al^2019^	60/30f	IMRT	291	OS:14.2mo	95
Carlson et al^2015^	PTV1:60Gy/10f PTV2:30Gy/10f	IMRT+TMZ+BEV	30	PFS:12.8mo OS:16.3mo	96
Carlson et al^2015^	PTV1:60Gy/10f PTV2:30Gy/10f	IMRT+TMZ	26	PFS:9.4mo OS:16.3mo	96
Zhong et al^2019^	64/27f	IMRT+TMZ	80	PFS:15mo OS:21mo	97
Reddy et al^2012^	60Gy/10f	IMRT+TMZ	24	OS:16.6mo	98
Monjazeb et al^2012^	70Gy/28f	IMRT	21	PFS:6.5mo OS:13.6mo	99
Jastaniyah et al^2013^	54.4Gy/20f	IMRT+TMZ	25	OS:15.67mo PFS:6.7mo	100

**Table 2:** Clinical trials of immunotherapy in HGG

Author	Target	Number of patients	Vaccine	Result	Reference
Cloughesy et al^2019^	PD1	16	neoadjuvant pembrolizumab	PFS:99.5d	101
	PD1	16	adjuvant nivolumab	PFS:72.5d	
Desjardins et al^2018^	CD155	61	PVSRIPO	OS:12.5mo	102
	--	104	--	OS:11.3mo	
Brown et al^2016^	IL13Rα2	1	IL13BBζ-CART	cytokines and immune cells in the cerebrospinal fluid increase	103
O'Rourke et al^2017^	EGFR	10	CART-EGFRvIII	OS:251d	104
Hilf et al^2019^	CD4/CD8	16	APVAC1/APVAC2	OS:29mo PFS:14.2mo	105

**Table 3:** Summary of studies for recurrent HGG treated with TTFields

Authors	Number of patients	Arms	OS (mo)	PFS (mo)	reference
Lazaridis et al^2020^	16	TTFields+TMZ +lomustine	--	20	91
Toms et al ^2019^	695	TTFields+TMZ	24.9	8.2	106
Stupp et al^2017^	466	TTFfields + TMZ (150-200 mg/m^2^/d for 5 days every 28 days for 6 cycles)	20.9	6.7	82
	229	TMZ alone	16	4	82
Kirson et al^2007^	10	TTFields (200kHZ, 2 V/cm,6d)	15.5	6.525	107
Kirson et al^2009^	10	TTFields (200kHZ,1.75V/cm,3d) +TMZ	>39	38.75	108
	10	TMZ	>14.7	7.75	
Stupp et al^2015^	210	TTFields+TMZ (150-200 mg/m^2^/d)	20.5	7.1	109
	105	TMZ	15.6	4	
Mrugala et al^2014^	457	NovoTTF	9.6	4.1	110
Stupp et al^2012^	117	TTFields (NovoTTF-100A)	6.6	2.2	111
	120	TMZ	6	2.1	
Ballo et al^2019^	148	TTFields(LMiFI >1.06 V/cm)	24.3	8.1	88
	192	TTFields(LMiFI<1.06 V/cm)	21.6	7.9	
Lu et al ^2019^	18	TMZ+bevacizumab+irinotecan+TTFields	18.9	10.7	92
	30	bevacizumab +TTFields	11.8	4.7	

## Abbreviations

CIRT: Carbon ion radiotherapy
CRT: Conventional radiotherapy
CTLA-4: Cytotoxic T-lymphocyte-associated protein 4
CTV: Clinical Target Volume
DC: Dendritic cell
EGFR: Epidermal growth factor receptor
GTV: Gross Target Volume
HFSRT: hypofractioned stereotactic radiotherapy
HGG: High-grade gliomas
IMRT: Intensity-modulated radiotherapy
LET: Linear energy transfer
MT: Microtubules
mTOR: mammalian Target of rapamycin
NCCN: National Comprehensive Cancer Network
OS: Overall survival
RBE: Relative biological effectiveness
PBT: Proton beam therapy
PD-1: Programmed cell death-1
PD-L1: Programmed death-ligand 1
PFS: Progress-free survival
PVC: Procarbazine, lomustine, and vincristine
SIB-IMRT: Simultaneous integrated boost intensity modulated radiotherapy
SRT: Stereotactic radiotherapy
TAMs: Tumor-associated macrophages
TGF-β: Transforming growth factor-β
TMZ: Temozolomide
TTFields: Tumor-treating fields
VEGF: Vascular endothelial growth factor
WHO: World Health Organization
3D-CRT: three-dimensional conformal radiotherapy
2D-CRT: two-dimensional conventional radiotherapy
